# Ibarapa Programme: Half a Century of Rural Health Service, Training, and International Cooperation in Nigeria

**DOI:** 10.1371/journal.pntd.0003201

**Published:** 2014-10-02

**Authors:** Oluwatoyin A. Asojo, Micheal C. Asuzu, Akindele O. Adebiyi

**Affiliations:** 1 Baylor College of Medicine, National School of Tropical Medicine, Houston, Texas, United States of America; 2 Ibarapa Programme, College of Medicine, University of Ibadan, Ibadan, Oyo State, Nigeria; Yale School of Public Health, United States of America

## The Ibarapa Programme: A Model of Rural Health Education

The Ibarapa Programme was founded on February 9, 1963, as the Ibarapa Project, a collaborative and cooperative health development project of the University of Ibadan, the Western Nigeria Government, and the Ibarapa community. The Rockefeller Foundation provided generous seed funding for the project, while the Liverpool School of Tropical Medicine and the London School of Tropical Medicine and Hygiene provided technical support to medical staff. The three specific objectives of the project as restated in the 25th anniversary book [1] were as follows:

- To teach medical students and doctors, through practical work, the principles and practices of community medicine
- To study the problems of health care delivery in the Ibarapa Community and to develop the health services of the district into a model of what an integrated local health service should be, in collaboration with the Government of Western Nigeria and in a manner that can be applied to other districts in Nigeria and other developing countries
- To carry out research into various aspects of health and disease in the community and thus to build up a body of knowledge on the various factors which are involved in health promotion and disease prevention

The Ibarapa Project evolved into the Ibarapa Programme and has remained an interdisciplinary training program in community health, development, and empowerment. The Ibarapa Programme has hosted several postings of resident doctors (at registrar and senior registrar levels), 217 community health postings of medical students (since 1963); six postings of dental students (from 2007); several 3-month internships for master's and diploma students from the African Regional Health Education Centre at the Department of Health Promotion and Education; over 21annual postings of community health officers in training at the University College Hospital (UCH); and many other postings of students from the UCH Schools of Nursing, Midwifery, and Perioperative Nursing, the Oyo State School of Midwifery, and the community health extension workers of the Oyo State School of Hygiene, Eleyele. In addition, elective students from the United States, Canada, the Netherlands, Britain, Finland, and other parts of the world come regularly to Ibarapa for experiences in community medicine and tropical medicine.

The impact of the Ibarapa Programme on health care in Nigeria is significant because a large number of practicing physicians in Nigeria are Ibadan-trained and spent eight weeks in the district, including one week at the District Hospital in Eruwa, where they learned the rudiments of secondary health care. The district hospital in Eruwa has won accolades in rural healthcare and surgery and in 2009 was the subject of the award-winning documentary *Innovating for Africa: Uncommon Service* [2]. Since 2007, all training in secondary health care has taken place at the Igbo Ora Comprehensive Health Centre of the Ibarapa Programme, with an expansion of the staff and facilities there, including the addition of a resident rural surgeon employed by the UCH, Ibadan [3]. The clinical medicine and health–related research is recorded in the 50th anniversary book. Studies in community leadership and development and regular internal evaluation and program improvement as well as ongoing activities are sometimes published in international literature [1]. The strategic involvement of several departments and faculties of the University of Ibadan (UI) from education, anthropology, sociology, geography, engineering, and health—has generated additional technology and data over the last 51 years, but there remains a need to develop modern searchable data repositories for the data.

Ibarapa Programme has collected 51 years of medical records of patients in a rural community and over 30 years of interdisciplinary data. The formation of the African Regional Health Education Center (ARHEC) in 1975 and subsequent training of health educators facilitated the generation of studies and papers from the Ibarapa Programme. ARHEC is an initiative that was started by the World Health Organization, the University of Ibadan, and the Nigerian Ministry of Health as a culturally competent and relevant health educational center for Africans [4]. Additionally, collaborators from all over the world have appreciated the value of the data and projects, using the data to help unravel the molecular and genetic basis of susceptibility to disease and infection. Health conditions that have been extensively studied in Ibarapa include multiple pregnancies, cancer, childhood malnutrition, snake bites, anemia, hypertension, dementia, glaucoma, medical entomology, divorce, adolescence-related health problems (including teenage pregnancy), hematological disorders (notably sickle-cell anemia), epilepsy, hernias, accidents, parasitic diseases, and backache [1]. Currently, the Ibarapa Programme is in need of funding, as well as training opportunities for local scientists and stakeholders in data management and storage for the continuous steam of data that is being generated. Key among the data is the repository of data on neglected tropical diseases that are endemic in the rural setting of the Ibarapa Programme.

## The Ibarapa Programme and Neglected Tropical Diseases (NTDs)

The Ibarapa Programme is located in a rural community in the lush tropical rain forests of southwestern Nigeria, and at initiation of the project, the area was endemic for many NTDs, notably intestinal parasites, onchocerciasis, and guinea worm [1], [5]. The rural home of the Ibarapa Programme is Igbo Ora, a rural town in Oyo State, Nigeria. In 1963, the population of Igbo Ora by the political de facto census was 30,000. The population had doubled by 2013 to 64,431, with children under age five accounting for 10.6% of this population [6]. Igbo Ora is 50 miles north of Lagos and is considered the twin capital of the world because of the unusually high number of twin births in the community. Igbo Ora is also the capital of the Ibarapa Central Local Government. Igbo Ora is a multiethnic community and home to the majority indigenous Yoruba and migrant minority groups including the Fulani cattle herders. As far back as 1979, the importance of the vital health statistic records collected by the Ibarapa Programme at Igbo Ora was recognized [7]. The health records have indicated births, deaths, incidents of illness, and other statistics on communicable and infectious diseases [8]–[11]. Additionally, a seminal paper in 1971 that recognized and identified leopard skin and other dermatological effects as manifestations of onchocerciasis came out of studies from the Ibarapa Programme [12]. The African Programme for Onchocerciasis Control (APOC) carried out several of its studies within the framework of the Ibarapa Programme [13]. Ibarapa Programme was also used as a site for the WHO/Carter Foundation–funded guinea worm eradication program [9], [14]. There is also ongoing research on intestinal parasites as well as other NTDs under the umbrella of the Ibarapa Programme [2]. Equally impressive is the generation of papers that reveal the medical status, stigma, and social and cultural implications of NTDs [1].

## Future Direction of the Ibarapa Programme

As the Ibarapa Programme turns 51 years old, the Comprehensive Health Centre at Igbo Ora and the public health program continue to grow, despite the upheavals that the country, the state, the community, and the university have faced. A major challenge facing the program is the lack of funding to adequately preserve and index the extensive health and other data that have been generated and continue to be generated. There is also a need for capacity building to train local stakeholders in the use of this extensive data, which is important for the eradication and understanding of NTDs and other diseases as well as of socioeconomic and cultural issues facing rural dwellers. Consequently, the Ibarapa Programme is undergoing major changes that will allow it to meet the current and future needs of the rural community and researchers.

**Figure 1:**
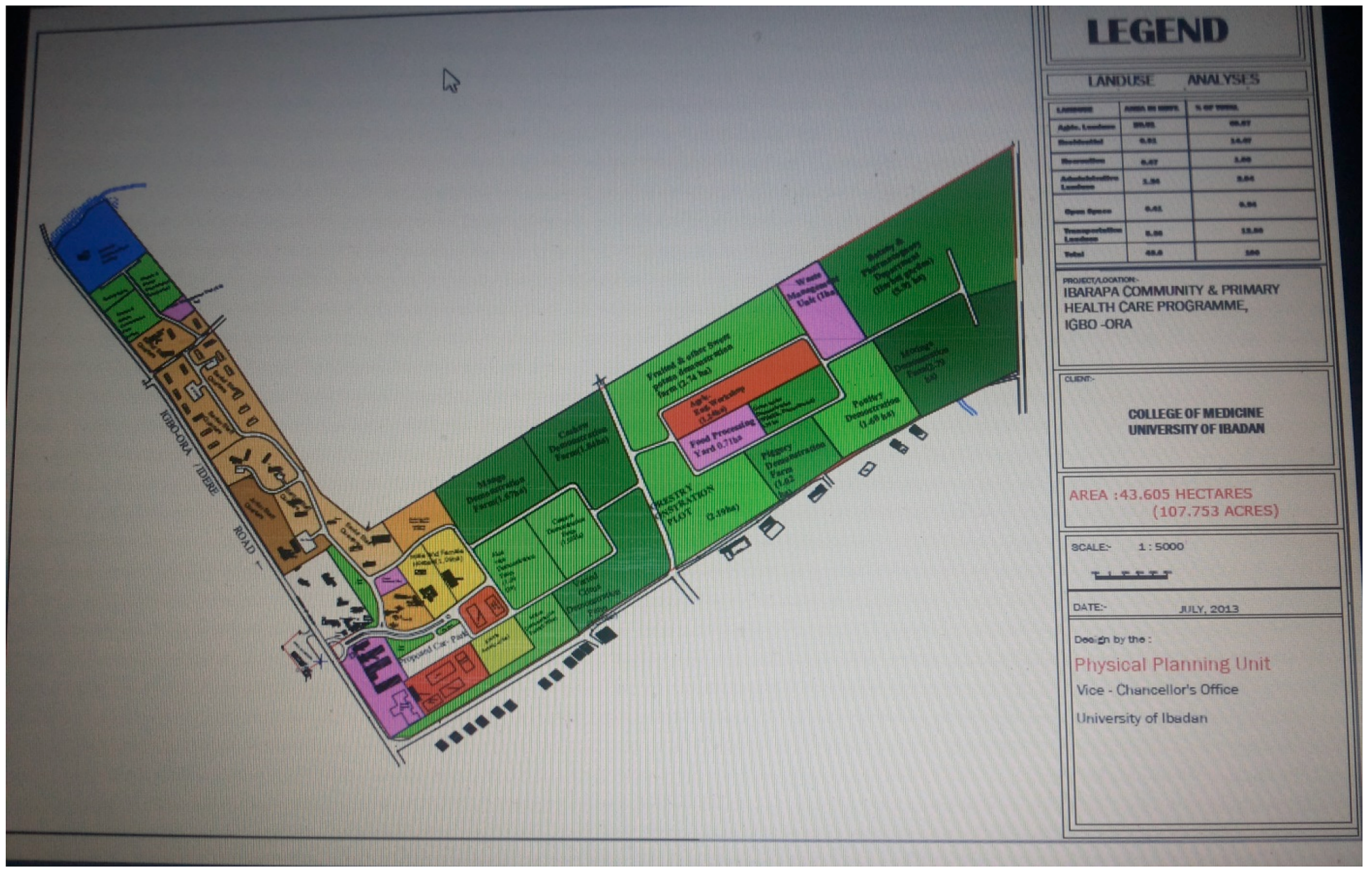
The integrated Ibarapa Programme master plan as produced by the Directorate of Physical Planning following a detailed soil survey of the land and consultations with the Ibarapa community and different university departments.

**Figure 2:**
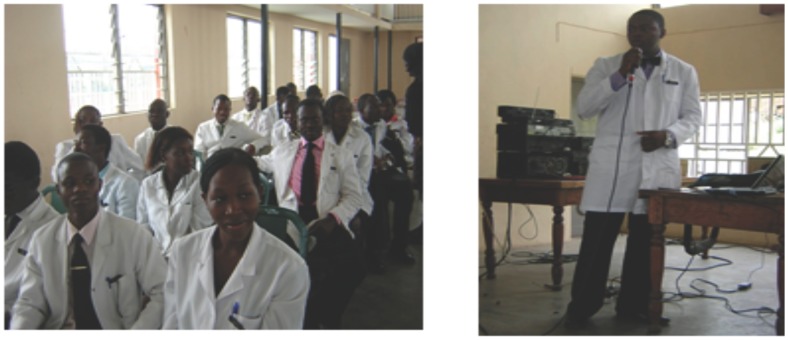
Medical students at a town hall presentation of their community health research at Eruwa Town Hall.

**Figure 3:**
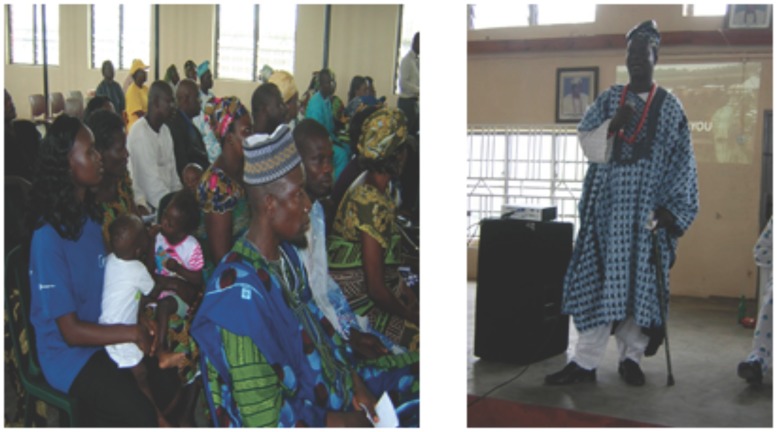
Community audience and one of their chiefs commenting on the policy implications of the findings of students' community health research at Eruwa Town Hall.

**Figure 4:**
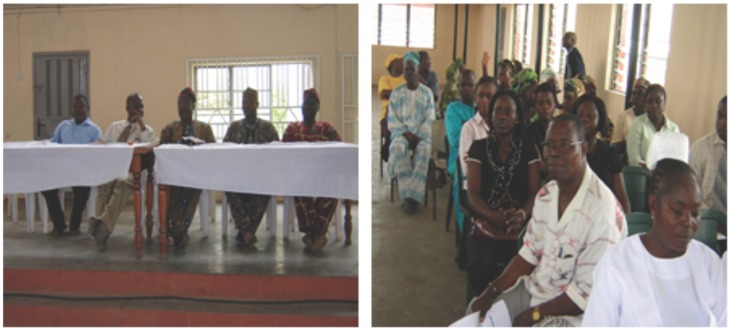
Health care workers and members of the local government area and community at an Eruwa Town Hall research-to-policy presentation of the students' research. Community and Programme leadership are at the high table.

A key change is the evolution of the Ibarapa Programme into the Institute of Community Health and Development (ICHD). The central offices of ICHD will be based at UCH, and its community development, research, and training program will involve all the faculties, departments, and units of UI. ICHD has been initiated with the urban and regional development of the Ibarapa zone through agriculture, conservation, and health forestry. Thus, the 43,605 hectares donated to the Ibarapa Programme in 1963, of which only 7.2 hectares have been used, has been subjected to detailed soil survey and agricultural characterization by the Department of Agronomy of the university. The master plan of the entire land was developed following a community needs assessment in conjunction with the local people, through town-hall exercises and community meetings (Figures 1–4). The ICHD is a rural development program that involves and trains students and stakeholders in all three of the Ibarapa local government areas (Figures 2–4). The ICHD students' research at Igbo Ora has been advanced to a defended prefield exercise, followed by the fieldwork and a postfield presentation of the implications of their findings at a joint community stakeholders and local authorities meeting termed “research to policy.” This is in addition to the scientific defense and write-up of the research itself.
